# 

**Published:** 1888-02-18

**Authors:** 


					I V\a *^l
vPRICE one penny.
*0. 73, VoL III-
February 18th, 1888.
(Registered at the General Post Office
as a Newspaper.]
u
Pjr Annum, 6s. 8cL. post free.
Monthly Parts, 6d.; post free, 7id,
Ditto per Ann., 7s- 6d. post free.
5;ieii:e, flft:btc(ne, arts (?Mlarttbcop2.

				

